# SHEAR: sample heterogeneity estimation and assembly by reference

**DOI:** 10.1186/1471-2164-15-84

**Published:** 2014-01-29

**Authors:** Sean R Landman, Tae Hyun Hwang, Kevin AT Silverstein, Yingming Li, Scott M Dehm, Michael Steinbach, Vipin Kumar

**Affiliations:** Department of Computer Science and Engineering, University of Minnesota, Minneapolis, MN USA; Quantitative Biomedical Research Center, University of Texas Southwestern Medical Center, Dallas, TX USA; Department of Clinical Sciences, University of Texas Southwestern Medical Center, Dallas, TX USA; Harold C. Simmons Cancer Center, University of Texas Southwestern Medical Center, Dallas, TX USA; Research Informatics Support Systems, Minnesota Supercomputing Institute, University of Minnesota, Minneapolis, MN USA; Masonic Cancer Center, University of Minnesota, Minneapolis, MN USA; Department of Laboratory Medicine and Pathology, University of Minnesota, Minneapolis, MN USA

**Keywords:** Genomics, Next-generation sequencing, Sequence analysis, Assembly, Personal genome, Heterogeneity, Structural variation, Prostate cancer

## Abstract

**Background:**

Personal genome assembly is a critical process when studying tumor genomes and other highly divergent sequences. The accuracy of downstream analyses, such as RNA-seq and ChIP-seq, can be greatly enhanced by using personal genomic sequences rather than standard references. Unfortunately, reads sequenced from these types of samples often have a heterogeneous mix of various subpopulations with different variants, making assembly extremely difficult using existing assembly tools. To address these challenges, we developed SHEAR (Sample Heterogeneity Estimation and Assembly by Reference; http://vk.cs.umn.edu/SHEAR), a tool that predicts SVs, accounts for heterogeneous variants by estimating their representative percentages, and generates personal genomic sequences to be used for downstream analysis.

**Results:**

By making use of structural variant detection algorithms, SHEAR offers improved performance in the form of a stronger ability to handle difficult structural variant types and better computational efficiency. We compare against the lead competing approach using a variety of simulated scenarios as well as real tumor cell line data with known heterogeneous variants. SHEAR is shown to successfully estimate heterogeneity percentages in both cases, and demonstrates an improved efficiency and better ability to handle tandem duplications.

**Conclusion:**

SHEAR allows for accurate and efficient SV detection and personal genomic sequence generation. It is also able to account for heterogeneous sequencing samples, such as from tumor tissue, by estimating the subpopulation percentage for each heterogeneous variant.

**Electronic supplementary material:**

The online version of this article (doi:10.1186/1471-2164-15-84) contains supplementary material, which is available to authorized users.

## Background

The last several years of genomics research has revealed the need to study whole genomic sequences, including regions that were once thought to be inconsequential, when trying to identify important individual genetic variations. A more thorough analysis of these variations requires the assembly of personal genomic sequences through resequencing. For instance, ChIP-seq analysis was recently shown to be more effective if aligned using a personal genome as opposed to a reference genome [1], and many other types of analyses, such as RNA-seq, may be aided by using personal genomic sequences as references for alignment.

**Figure 1:**
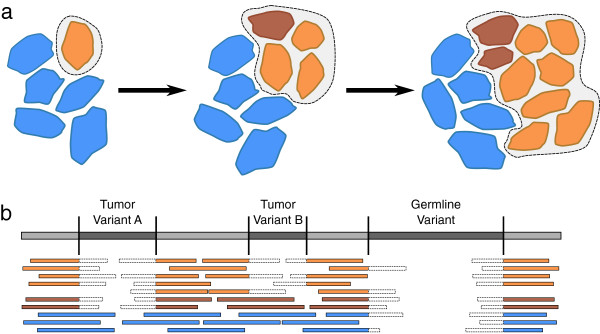
**Variants in tumor DNA can result in heterogeneous alignment patterns in tumor samples.** (**a**) The progression of tumor development illustrates how neighboring cells can have several different genomic sequences. The tumor cells (orange) originate with a genetic mutation in one cell and grow rapidly to outnumber the normal cells (blue). During the rapid growth of the tumor, additional genomic variants are created through further mutations (brown), resulting in a heterogeneous mix of normal and multiple tumor genomic sequences. (**b**) Reads sampled from the normal cells (blue) and two subpopulations of tumor cells (orange/brown) are aligned against the reference sequence. Soft-clipped portions are indicated by a dotted line border. A germline-acquired deletion is present in all of the sample, but deletions present in only the tumor cells result in some reads being aligned to the reference and others being soft-clipped. Disregarding the possibility of heterogeneous sequencing samples might result in these variants being missed.

In many cases, personal genome assembly is also complicated by sample heterogeneity, such as tumor samples with subpopulations of somatically-acquired variants that are present in only a subset of the sequencing data. Genomic instability and rapid division of cancer cells can lead to a high degree of cellular heterogeneity within tumor tissue. Sequencing data from tumor samples will usually contain mixtures of DNA from diverse tumor cell populations, as well as from normal somatic cells, each with their own set of variants. The assembly programs currently used to create personal genomic sequences often assume sample homogeneity and are plagued by an inability to handle non-universal variants (see Figure 1), but these tools must account for this type of data if they are to be useful for studying cancer genomics.

If there is a closely-related reference genome available, alignment-based assembly is a simplistic way to perform assembly of personal genomes. The sequenced reads are aligned to the reference genome using a short-read aligner such as BWA [2] or Bowtie [3], and SNPs and small indels are determined by consensus amongst the aligned reads. Although this is usually efficient, complete personal genomic sequences must also account for the presence of structural variation (i.e. deletions, insertions, duplications, inversions, or translocations of large segments of DNA). The opposite extreme of approaches is to join together all the sequenced reads, like pieces of a puzzle, by determining how they overlap with one another. This class of assembly algorithms, known as *de novo* assembly, does not require a reference sequence and is useful for assembling regions that are significantly different from the available reference genome, such as novel insertions. However, *de novo* assembly may struggle to properly assemble repetitive regions and can be extremely inefficient at the high coverage levels often required for assembling whole genomes. Examples of global *de novo* assembly algorithms include Velvet [4], SOAPdenovo [5], and ALLPATHS-LG [6].

Algorithms have recently been developed that combine aspects of both alignment-based assembly and *de novo* assembly. Seq-Cons [7] and LOCAS [8] use localized versions of *de novo* assembly in order to assemble reads in separate blocks determined by the area of the reference that they are first aligned to, rather than trying to determine possible overlaps between all of the reads globally, without a preliminary alignment. A similar approach was also shown to be successful in assembling several variant strains of *Arabidopsis thaliana* from a related reference genome [9]. RACA [10] uses a reference genome to arrange the scaffolds that are first produced through *de novo* assembly, but also requires multiple outgroup genomes (i.e. from other closely related species) as input.

In contrast to the above methods that can be thought of as either "global *de novo* assembly followed by alignment" or "alignment followed by local *de novo* assembly", IMR/DENOM is a reference-guided assembly approach that combines alignment-based assembly and *de novo* assembly in parallel and merges the results [11]. The alignment-based half of the algorithm, IMR, is an iterative procedure that creates an alignment to the original reference sequence using Stampy [12], generates a new reference sequence from consensus variants in the alignment, realigns the paired-end reads to the new reference sequence, and repeats this procedure until convergence. DENOM takes contigs that are assembled *de novo* using SOAPdenovo [5] and aligns them to the reference in order to handle larger SVs, such as novel insertions not present in the reference sequence. The results of these two approaches are then merged to generate a personal genomic sequence.

All of these assembly programs assume sample homogeneity, resulting in unexpected behavior when sequencing samples contain variants only present in subpopulations of the cells, and thus will struggle with the type of tumor data described previously. Certain types of SVs, such as tandem duplications, also remain a challenge. Additionally, these approaches are often inefficient in the context of personal genome assembly due to the large amount of redundant operations performed, such as the multiple alignments of every read in IMR, or the assembly of every read in reference-guided assemblers such as Seq-Cons or LOCAS. A more efficient approach for generating personal genomic sequences might be to leverage the specialized ability of pre-existing SV detection programs to locate individual SVs and address them directly, rather than hoping to discover their signature through *de novo* assembly (which is made more difficult in the presence of heterogeneous sequencing samples). A variety of SV detection algorithms have been developed over the last few years [13–18] which can serve for this purpose.

In this paper, we propose a new tool called SHEAR (Sample Heterogeneity Estimation and Assembly by Reference). SHEAR creates personal genomic sequences by predicting SVs and generating the new genomes based on the existing reference after correcting for those SVs, while also offering the ability to handle heterogeneous sequencing samples. Our novel contributions come in the form of two key concepts that integrate and expand several pre-existing programs, namely a pipeline to improve alignments (and thus subsequently improve SV prediction) by correcting errant soft-clipping at probable SV breakpoints, and a scheme to estimate heterogeneity percentages for SV predictions for a variety of SV types. The level of heterogeneity for SVs present in the sequencing sample is estimated by comparing soft-clipped and spanning reads at the SV breakpoints, allowing users to generate the genomic sequences of the particular sample subpopulations they are interested in. In contrast to using existing *de novo*, alignment-based, or reference-guided assembly methods for generating personal genomic sequences, SHEAR is able to handle heterogeneous sequencing data, has a better ability to detect tandem duplication events, and offers improved computational efficiency by focusing on known variant regions and reducing repetitive operations in concordant regions. We compare our method against existing approaches on a variety of simulated data sets as well as real sequencing data from a prostate cancer cell line with known heterogeneous variants.

## Implementation

Unlike *de novo* assemblers or the iterative alignment-based assembly done by IMR, our approach works by doing a one-time global alignment which is used to predict SVs and correct them directly to create a personal genomic sequence. Candidate SV regions are refined through further local alignment, and finally SNPs and small indels are determined using an alignment-based assembly once the genomic sequence is structurally similar and reads that span SV breakpoints are properly aligned. Heterogeneity percentages for SVs are estimated using a novel scheme that compares counts of soft-clipped and spanning reads at SV breakpoints in a variety of ways, depending on the SV type.

SVs are predicted from the alignment using CREST [15], an SV prediction algorithm that uses the split-read approach. Split-read approaches look at reads that are only partially aligned to the reference, with the remainder being "soft-clipped". This methodology discovers SVs by finding pairs of soft-clip clusters that match each other in the reference, indicating an adjacency of genomic regions that are normally separated in the reference. CREST was selected for this purpose because it predicts breakpoints at base pair resolution, handles many different SV types (with good support for tandem duplications in particular), and is designed for somatically-acquired variants as well as germline variants. However, SHEAR is designed so that any base pair resolution SV detection algorithm could be substituted in to accomplish this task. Thus, as SV detection algorithms improve in accuracy and efficiency, so too will SHEAR.

**Figure 2:**
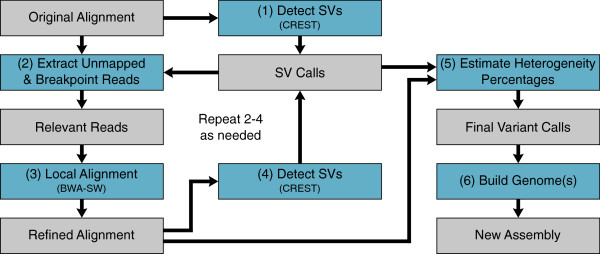
**SHEAR workflow diagram.** (1) SHEAR’s workflow begins by using CREST to predict the locations of SVs from the original SAM/BAM alignment file. (2) Reads neighboring the breakpoints of the predicted SVs, as well as all unmapped reads, are extracted from the original alignment. (3) These extracted reads are then realigned using a local alignment algorithm (BWA-SW) to improve the soft-clipping accuracy near the breakpoints. Breakpoint extracted reads are aligned in their original neighborhoods, and unmapped extracted reads are aligned against the whole reference sequence. (4) SVs are again predicted from the new alignment, which contains only the realigned reads near the original candidate breakpoints, as well as reads that were initially unmapped but have been realigned using the local alignment algorithm. The new SV predictions will potentially include new SVs and refined breakpoints of previously predicted SVs. Steps 2–4 may be repeated as necessary to pick up new SV events, and will usually only need to be repeated 2–3 times before SV predictions remain constant. (5) Using the refined predicted SV breakpoints, the heterogeneity percentage of each SV is estimated by comparing the soft-clipped and spanning reads at the breakpoints. This calculation varies depending on the SV type (see Figure 4). (6) Finally, a new personal genomic sequence is created by using the predicted SVs to directly modify the original reference sequence.

There are two key novel components to the SHEAR framework. The first is a pipeline for correcting soft-clipping errors in the alignment that occur at the breakpoints of candidate SVs. Although these alignment errors are often minor, correcting for them improves the reliability of CREST predictions, as well as the accuracy of our heterogeneity estimation. Thus, the first component of our proposed method enables and enhances the second component, which is a novel scheme for estimating the heterogeneity of predicted SVs of various types. This is done by comparing the soft-clipped reads to the spanning reads at SV breakpoints. The following two sections give a more detailed description of these two components of the SHEAR algorithm, and an illustration of SHEAR’s overall workflow can be see in Figure 2.

Although the bulk of this paper focuses on heterogeneity estimation and improving SV predictions by refining soft-clipping at candidate breakpoints, the other main purpose of SHEAR is to generate personal genomic sequences by modifying the reference sequence using the most common set of predicted variants, or the set of variants chosen by the user. Future versions will automate the generation of multiple personal genomic sequences using phasing information derived from the estimated heterogeneity levels. The SV prediction and heterogeneity estimation component of SHEAR can also be run independently of the component to generate personal genomic sequences. Our program is implemented using the Genome Analysis Toolkit (GATK) framework [19] to provide efficient data access and for ease of parallelization, with additional tools used from SAMtools [20] and Picard [21].

### Pipeline for improving SV predictions by refining soft-clipping at candidate breakpoints

Paired-end reads are first aligned to the original reference sequence with BWA [2] using default parameters. Polymerase chain reaction (PCR) duplicates are removed using Picard [21]. This produces an alignment on the whole sequence that will be referred to as the original alignment. An initial set of SVs are then predicted by CREST, each of which is characterized by an SV type (i.e. duplication, deletion, etc.) and two breakpoint locations in the genome.

Both the CREST prediction accuracy and our estimation of heterogeneity percentage depend on having an accurate alignment in which reads sampled from a variant subpopulation are properly soft-clipped at that subpopulation’s SV breakpoints. Unfortunately, this is not always the case for an alignment done using BWA. If a sufficient global alignment is not found for an aligned read’s pair, BWA will perform local alignment in the genomic region near where the read’s pair is predicted to occur. This local alignment algorithm will soft-clip any ends that do not align.

**Figure 3:**
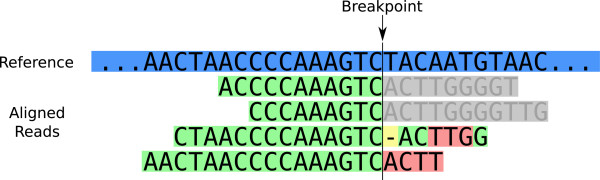
**Soft-clipped reads vs. improperly aligned reads at a structural variant breakpoint.** The reference sequence is indicated in blue. Green bases are alignment matches, pink bases are mismatches, yellow bases are skips, and gray bases are soft-clipped. All four reads are sampled from the variant sequence and span the breakpoint, but only the top two are properly soft-clipped. The bottom two reads are aligned with mismatches and skips because the portion that should be soft-clipped is only a few bases long and the global alignment is used instead. Inaccurate soft-clipping can lead to false negatives for split-read based SV prediction algorithms and also lowers the accuracy of SHEAR’s heterogeneity estimation algorithm.

However, many reads that should be soft-clipped are missed for two reasons. First, reads that are almost fully aligned to the reference sequence (i.e. only a few bases extend past the breakpoint) can be aligned with mismatches in the global alignment portion of the BWA algorithm. In this situation, local alignment is not attempted because the global alignment is sufficient, and thus there is no soft-clipping done. An example of this can be seen in the bottom two aligned reads in Figure 3. Second, reads that should be soft-clipped at a breakpoint might remain unmapped completely. This occurs when BWA determines that neither the global alignment nor the local alignment are of sufficient quality. In both cases, the soft-clipped local alignment represents the "true" alignment, but the aligner has no way of knowing the location of the SV breakpoint, and thus will instead force a mismatched global alignment or leave the read unmapped.

These issues could potentially be addressed by modifying BWA’s parameters to be more stringent for accepting global alignments and less stringent for soft-clipped local alignments. However, this can introduce additional issues when aligning other concordant reads. Assuming we have at least some reads from variant breakpoints that are aligned with the correct soft-clipping, we can use the predicted SVs to hone in on the exact areas that are likely to experience the above problems and correct them.

To address these problems, we remap these reads using the BWA-SW algorithm for local alignment [22]. BWA-SW is less efficient than the global alignment of the regular BWA algorithm and can only align reads individually, not in pairs, but it does allow for soft-clipping rather than requiring the entire read to be aligned. We account for this inefficiency by remapping only reads that might be affected by the two issues discussed above. Specifically, all unmapped reads, as well as reads that align either spanning or soft-clipped at one of these breakpoints, are extracted from the original alignment. This collection of reads are then realigned with BWA-SW using default parameters to produce a new alignment. Leaving the concordantly aligned reads alone improves the efficiency of our approach in comparison with *de novo* assemblers or the iterative alignment of IMR. Aligning these reads individually removes the pairing restrictions that may lead to unmapped reads when using default BWA, as discussed above.

The corrected version of the alignment is then passed back to CREST to make revised SV predictions. New SV predictions can be picked up if there were not a significant number of supporting soft-clipped reads in the original alignment. At this point, the unmapped and breakpoint reads can be extracted again from the original alignment to correct the soft-clipping at these new SV locations, using the same procedure. In practice, we observe that this rarely needs to be repeated more than three times before SV predictions remain unchanged. Finally, before estimating heterogeneity percentages for each SV, SHEAR will remove some variants that are slight derivations from other predicted SVs (i.e. only differing by a few base pairs on one breakpoint). If the differing breakpoint has only a few supporting soft-clipped reads in comparison with a "main" predicted breakpoint with many more, it is usually due to sequencing error and thus can be discarded as a false positive.

### Using soft-clipped reads to estimate variant heterogeneity

**Figure 4:**
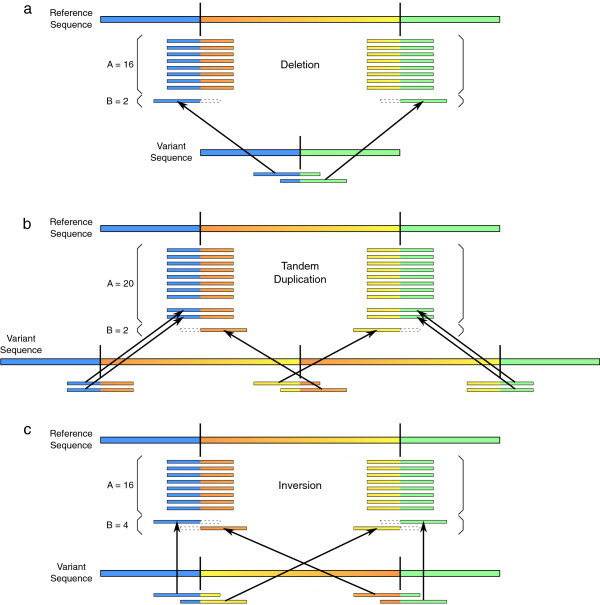
**Alignment of reads from a heterogeneous sequencing sample.** Total counts of soft-clipped reads and spanning reads at the structural variation breakpoints are different between the cases of (**a**) deletion, (**b**) tandem duplication, and (**c**) inversion. All examples have 10 × depth of coverage, with 20% of the sample coming from the variant sequence (i.e. 2 × depth) on the bottom and 80% from the reference-like sequence (i.e. 8 × depth) on top. Arrows indicate how reads from the variant sequence will be aligned against the reference genome, with soft-clipping indicated by dotted line borders. *A* indicates the total number of reads that span either breakpoint, and *B* indicates the total number of reads that are soft-clipped at either breakpoint in each of the examples. *A* and *B* are used by SHEAR to estimate the heterogeneity of SVs.

After no additional SV predictions are picked up by CREST, the heterogeneity percentages for each SV are estimated by comparing the average number of reads per locus from the reference-like sequence (estimated reference depth (*R*)) with the number of reads per locus from the variant sequence (estimated variant depth (*V*)). These can be estimated from the numbers of reads that span the SV breakpoints and the number of reads that are soft-clipped at these breakpoints, respectively. The way this calculation is done depends on the type of SV. In all cases, the goal is to determine the estimated reference depth (*R*) and the estimated variant depth (*V*), and to estimate the heterogeneity percentage by comparing the two. Let the total number of aligned reads that span either breakpoint be *A*, and the total number of reads that are soft-clipped at either breakpoint be *B*. Figure 4 illustrates the examples for deletions, tandem duplications, and inversions. In these examples, the variant subpopulation is always 20% of the sample.

In the case of a deletion, the original reference has two breakpoints that are merged into the same locus in the variant subpopulation (Figure 4a). Reads sampled from the variant subpopulation that span this breakpoint will align (with soft-clipping) to either of the corresponding breakpoints in the reference, usually depending on which half of the read the breakpoint is at. Thus, the estimated variant depth (*V*) is the total number of reads soft-clipped at the two reference breakpoints (*B*). The estimated reference depth (*R*) is taken from the average number of spanning reads between the two breakpoints, ( ). The heterogeneity percentage, *H*, is estimated as:


In the example in Figure 4a, *A* = 16 and *B* = 2, giving an estimated variant depth of 2 × and an estimated reference depth of , for a 20% estimated heterogeneity.

The case of a tandem duplication is slightly more complicated (Figure 4b). Reads from the variant subpopulation that span the new fusion (i.e. the middle of the tandem repeat) will again map to either of the two reference breakpoints depending on which side of the breakpoint each is more aligned with. However, reads that align correctly to the outside edges of the duplicated segment could have been sampled from either the reference-like sequence or the variant sequence. In order to get an accurate estimate of the number of reads from the reference-like sequence, we must account for the fact that *A* contains reads from both populations. Again we take the total number of soft-clipped reads at both breakpoints, *B*, as the estimated variant depth. This is then subtracted from the average number of spanning reads to arrive at an estimated reference depth. The heterogeneity percentage is thus estimated as:


In the example in Figure 4b, *A* = 20 and *B* = 2, giving an estimated variant depth of 2 × and an estimated reference depth of , for a 20% estimated heterogeneity.

Unlike deletions and tandem duplications which have a difference in the number of breakpoints involved between the reference-like sequence and the variant sequence, inversions have two breakpoints in each sequence (Figure 4c). Reads sampled from the variant subpopulation that span these breakpoints will again map to either of the reference breakpoints, depending on their location. Because there is no copy number change in an inversion, we can directly estimate the heterogeneity by comparing the total number of soft-clipped reads with the total number of spanning reads at the two breakpoints of the inversion. Heterogeneity is thus estimated as:


For this example (Figure 4c), *A* = 16 and *B* = 4, and a direct estimation of heterogeneity is again 20%.

Note that the above calculations for *H* all assume intra-cellular homozygosity (i.e. all copies of the genetic locus within the cell contain the variant), but the likelihood of heterozygosity in real-world data sets should be considered when interpreting the meaning of *H*. Thus, a reported heterogeneity estimation of *H* = 50*%* could imply either that 100% of the cells in the sequencing sample are heterozygous for the variant in a diploid case, or that 50% of the cells in the sequencing sample are homozygous for the variant.

## Results and discussion

To evaluate our methods, we evaluated SHEAR on simulated data sets as well as a prostate cancer cell line sample with validated heterogeneous variants. We compare our results with IMR/DENOM [11] to demonstrate the advantages of our approach, namely, improved computational efficiency, better support for tandem duplications, and the ability to handle personal genome assembly in the presence of heterogeneous sequencing samples as well as to estimate the level of that heterogeneity. IMR/DENOM alone was chosen for comparison because other reference-guided assembly methods produce contigs or are generally designed for creating new reference sequences rather than "personalizing" existing reference sequences, which is the purpose of SHEAR.

### Simulated data

A reference sequence to be used for simulation was taken from a 70 kbp region of chromosome 15 (25,420,001-25,490,000) chosen because of its non-repetitiveness. The length of this sequence is intended to be on the scale of the size of a long gene. Twenty different variant sequences are then created by introducing ten different sets of deletions and ten different sets of tandem duplications at known locations. These represent two SV types easily handled by CREST. Other SV types could be handled by incorporating additional SV predictors. Each set of SVs contained three non-overlapping SVs of sizes 150 bp, 1000 bp, and 30 kbp.

Sequencing simulation was then performed on each of the variant sequences by randomly sampling paired-end reads, with read lengths of 75 bp and fragment sizes sampled from a truncated normal distribution with a mean of 250 bp, standard deviation of 20 bp, inclusive lower bound of 175 bp, and inclusive upper bound of 325 bp. For each sequencing simulation, a portion of the paired-end reads were sampled from the original sequence as well as from the variant sequence. This heterogeneity percentage was varied (20%, 40%, 60%, 80%, 90%, and 100% from the variant sequence), as was the overall average coverage (10 ×, 20 ×, 30 ×, 50 ×, 100 ×, 500 ×, and 1000 ×). There were no synthetic sequencing errors and base quality was reported as perfect (i.e. phred score of 40). This was to eliminate the effects of sequencing errors in order to evaluate the two methods solely on their algorithmic approach. These simulated data sets were intended to be easy to handle, in order to control for issues with fragment size distributions, sequencing errors, SNPs, small indels, and cross-chromosomal events. Instead, we focus specifically on the issue of how to account for heterogeneous SVs.

Using a 20 × overall coverage and varying the portion of simulated reads that originated from the variant sequence (Table 1), our method demonstrates a strong ability to handle heterogeneous SVs. IMR/DENOM is only able to reliably detect deletions in relatively homogeneous sequencing samples. Even at very high overall coverage (i.e. 1000 ×), IMR/DENOM is still unable to pick out heterogeneous variants, suggesting that this is not due to a lack of supporting reads (see Table S1 in Additional file 1). Tandem duplications are never identified using IMR/DENOM with our simulated data.

**Table 1:** **Correctly detected SVs for simulated data at 20 × coverage under varying levels of heterogeneity**

	Deletions		Tandem duplications	
Variant percent	SHEAR	IMR/DENOM	SHEAR	IMR/DENOM
**20%**	6 / 30	0 / 30	3 / 30	0 / 30
**40%**	20 / 30	0 / 30	14 / 30	0 / 30
**60%**	26 / 30	0 / 30	24 / 30	0 / 30
**80%**	29 / 30	1 / 30	26 / 30	0 / 30
**90%**	29 / 30	1 / 30	24 / 30	0 / 30
**100%**	28 / 30	27 / 30	26 / 30	0 / 30

All simulations are done on a 70,000 bp portion of chromosome 15 after introducing deletions and tandem duplications of sizes 150 bp, 1000 bp, and 30,000 bp, each over 10 different iterations, for a total of 30 different deletion events, and 30 different tandem duplication events.

Table 2 demonstrates our method’s ability to scale down to lower coverage levels even in the heterogeneous case where only 20% of the reads are sampled from the variant sequence. IMR/DENOM fails to detect any of the SVs present in the sample, while our method scales down well to 50 × overall coverage and even picks up a few events from the 30 × and 20 × coverage data sets. The depth of coverage required to detect SVs depends on the heterogeneity percentage of each SV. For example, as seen in Table 2, 20 × coverage is too low to reliably pick up variants that only comprise 20% of the sequencing sample. However, the same coverage level was enough to detect most of the variants (50/60) simulated at 60% heterogeneity level (see Table S2 in Additional file 1).

**Table 2:** **Correctly detected SVs for simulated data at 20% heterogeneity under varying levels of coverage**

	Deletions		Tandem duplications	
Depth	SHEAR	IMR/DENOM	SHEAR	IMR/DENOM
**10 ×**	1 / 30	0 / 30	0 / 30	0 / 30
**20 ×**	6 / 30	0 / 30	3 / 30	0 / 30
**30 ×**	13 / 30	0 / 30	13 / 30	0 / 30
**50 ×**	21 / 30	0 / 30	21 / 30	0 / 30
**100 ×**	29 / 30	0 / 30	28 / 30	0 / 30
**500 ×**	29 / 30	0 / 30	28 / 30	0 / 30
**1000 ×**	29 / 30	0 / 30	28 / 30	0 / 30

All simulations are done on a 70,000 bp portion of chromosome 15 after introducing deletions and tandem duplications of sizes 150 bp, 1000 bp, and 30,000 bp, each over 10 different iterations, for a total of 30 different deletion events, and 30 different tandem duplication events.

Although CREST is used as the underlying SV detection algorithm, SHEAR’s pipeline for correcting soft-clipping errors in the alignment via targeted local realignment improves the SV predictions in comparison to using CREST alone. Of the 2520 SVs in the simulated data set (i.e. twenty different variant sequences × three SVs per variant sequence × seven different coverage settings × six different heterogeneity percentage settings), 2072 were predicted by both CREST and SHEAR, but SHEAR improved the accuracy of breakpoint prediction for 502 (24.22%) of these, whereas CREST only improved the breakpoint accuracy for three SVs (see Table S3 in Additional file 1). Additionally, SHEAR’s local realignment component tends to increase the number of supporting soft-clipped reads for each predicted SV, with a 7.26% relative increase of supporting reads for each SV prediction.

Finally, the novel benefit of our approach is the estimation of heterogeneity of discovered SVs. Table 3 demonstrates the consistent accuracy of our heterogeneity estimation on SVs that are discovered. As expected, there is more error in estimating the heterogeneity level of tandem duplications than there is for deletions due to the heuristics used to estimate the number of reads from the reference-like sequence (see Figure 4b). The average error of heterogeneity estimation is higher at lower coverage levels, due to the smaller sample size of reads, and this effect is amplified for the more difficult problem of estimating the heterogeneity of tandem duplications. SHEAR’s local realignment component also helps to improve the accuracy of heterogeneity estimation by fixing incorrectly soft-clipped reads. In comparison with estimating heterogeneity from the original alignment, heterogeneity estimation is improved by an average of 11.26 percentage points across the 2520 SVs in the simulated data set after SHEAR’s targeted local realignment.

**Table 3:** **Average error of heterogeneity estimation**

	Deletions			Tandem duplications		
Depth	150 bp	1000 bp	30 kbp	150 bp	1000 bp	30 kbp
**10 ×**	0.00	1.22	1.46	13.98	13.73	12.27
**20 ×**	0.08	0.52	1.14	8.91	11.22	12.40
**30 ×**	0.17	0.42	1.06	9.64	11.48	11.86
**50 ×**	0.10	0.27	1.17	9.83	9.69	9.20
**100 ×**	0.08	0.21	1.21	5.56	5.47	6.06
**500 ×**	0.09	0.21	1.04	4.17	2.35	4.09
**1000 ×**	0.08	0.18	1.06	4.58	2.11	3.40

Each entry reports the average absolute error for estimation of heterogeneity percentage for a variety of SVs at different overall coverage levels. For each pairing of SV type and coverage level, ten iterations of simulation were sampled from each of seven different underlying heterogeneity percentages (20%, 40%, 60%, 80%, 90%, and 100%) for a total of 70 simulations per entry in the table. The reported error is the absolute difference between SHEAR’s estimation of heterogeneity percentage and the true percentage of breakpoint reads originating from the variant sequence. Each entry in the table contains the average error for that scenario, ignoring simulations in which the SV was not predicted. For example, for the first entry in the table (150 bp deletion at 10 × depth), the SV is only predicted in 24 out of the 70 simulations due to the low coverage, and thus the average error is from those 24 estimations.

### Tumor cell line data

To evaluate our methods with an experimental data set, we used next-generation sequencing data from previous work that examined the role that variants in the androgen receptor (*AR*) gene have on castration-resistant prostate cancer (CRPCa) [23]. Paired-end reads were sampled at 6000 × coverage from non-repetitive regions of the *AR* locus in genome DNA from the CWR-R1 cell line model of CRPCa. Reads were 76 bp in length with a 208 bp median fragment size (62.44 bp standard deviation).

The entire SHEAR pipeline (including initial alignment using BWA) completed execution in just under 16 hours using a cluster of eight Intel Xeon 2.66 GHz processors. The two components of IMR/DENOM could be run in parallel, with IMR, the more computationally expensive component due to the iterative alignments, taking more than three days on 24 Intel Xeon X7542 "Westmere" 2.66 GHz processors. These results indicate that SHEAR offers an efficiency advantage over IMR even though both operate iteratively, because SHEAR excludes concordantly aligned reads from future iterations. For example, the first execution of CREST in the SHEAR pipeline took seven hours, whereas the two subsequent executions of CREST took less than an hour combined.

Table 4 lists the results found from this data set. SV #1 is a translocation between the *GALK2* gene locus on chromosome 15 and intron 1 of *AR*. SVs #2-4 are deletions within intron 1 of *AR* while SV #5 is located in intron 2. SV #6 is a deletion of the exact locus of intron 6 in *AR* and thus is likely the result of cDNA copies of mRNA present in the sequencing sample, as this sample was prepared in a lab that frequently works with *AR* expression vectors. Thus the supporting reads for this SV call might have come from cDNA containing exons 6 and 7 spliced together. None of the predicted SVs were found by IMR/DENOM because of the heterogeneity of the sample, highlighting the advantage of using SHEAR on heterogeneous sequencing samples.

**Table 4:** **Results from the CWR-R1 cell line data**

No.	SV type	Breakpoints		Variant percent
**1**	Translocation	chr15:49,498,516	chrX:66,829,481	1.42%
**2**	Deletion	chrX:66,812,839	chrX:66,861,669	29.21%
**3**	Deletion	chrX:66,813,091	chrX:66,861,564	1.73%
**4**	Deletion	chrX:66,830,140	chrX:66,861,904	1.68%
**5**	Deletion	chrX:66,874,605	chrX:66,896,916	0.76%
**6**	Deletion	chrX:66,941,805	chrX:66,942,669	0.36%

Left and right breakpoint locations on the reference sequence are given for each predicted structural variation, as well as the estimated levels of heterogeneity.

SVs #2, #3, #4, and #5 were experimentally validated in this cell line sample using nested polymerase chain reaction (PCR) with deletion-spanning primers to amplify candidate SV regions, and Sanger sequencing to verify the joined sequences. We validated SV #3 in a previous study [23], and the PCR gel enrichments and electropherogram peak traces for SVs #2, #4, and #5 clearly confirm their presence in the sequencing sample (see Figure S1 in Additional file 1). Additionally, SHEAR removed eight CREST predictions that were slight derivations of these four reported SVs and that SHEAR determined to be false positives due to sequencing error. The experimental validation of SHEAR’s reported SV breakpoints confirms that the removed CREST predictions were indeed false positives. We were unable to validate SV #1 via nested PCR, but both of its breakpoints are located in repeat regions of the genome, making validation more difficult. This result could be spurious, however, the PCR validation of SVs #3, #4, and #5 suggests that SHEAR has the capability to identify true variants present in a very small percentage of the sample.

SVs #1 and #6 were not predicted by running CREST alone, outside of the SHEAR framework. It is only by re-running CREST after performing our pipeline to fix soft-clipping errors that there is enough evidence to successfully detect these two SVs. As mentioned, we were unable to validate SV #1 via nested PCR, and SV #6 is believed to be the result of RNA contamination. However, even though SV #6 is not an SV of interest, it still likely represents a true event in the sample and thus demonstrates how the SHEAR pipeline can improve upon using CREST alone. Additionally, for the other SVs, the SHEAR pipeline improves the confidence of the CREST prediction by increasing the number of soft-clipped reads that are concordant with the breakpoint pairs. For example, SV #2 is supported by 773 soft-clipped reads after running CREST on the default alignment, but has 1114 supporting reads using the SHEAR pipeline.

In our previous work, we also determined that there is a 20–30% decrease in copy number in the region of these deletions using multiplex ligation-dependent probe assay (MLPA) [23]. Previously thought to be attributed to a subpopulation with SV #3, this is instead precisely consistent with the heterogeneity level of the deletion of SV #2, as estimated by SHEAR. At the left breakpoint of SV #2, there are 702 reads that are soft-clipped and 3,094 reads that span the breakpoint, while the right breakpoints has 412 soft-clipped reads and 2,306 spanning reads (see Figure S2 in Additional file 1). Using SHEAR’s heterogeneity estimation scheme described earlier, for this SV we would have *A* = 3,094 + 2,306 = 5,400 and *B* = 702 + 412 = 1,114 for an estimated heterogeneity of:


Deletions in this region have been implicated in alternative splicing of the *AR* gene, which can result in resistance to androgen depletion therapy (ADT) in CRPCa patients.

It should be noted that there is a natural bias towards sampling DNA that is more similar to the reference sequence when doing targeted sequencing due to the "baits" used to target specific regions for sequencing. Divergent genomic sequences will be less likely to be sampled, especially when the baits are close to the breakpoints. This bias would be present in this tumor cell line data, meaning that our calculated heterogeneity percentages are likely underestimated by an unknown amount. The agreement between our computational estimation and the MLPA wet-lab estimation suggests that this underestimation is small, however it is still present nonetheless.

### Tumor whole-genome sequencing data

For both the simulated and cell line data sets, the sequencing data analyzed is sampled from genomic regions on a gene-size scale (i.e. tens or hundreds of kbp). To demonstrate SHEAR’s ability to scale up to larger data sets, we ran SHEAR on whole-genome sequencing data from liver metastatic tumor tissue from a lung cancer patient [24]. This data set had an average depth of coverage of 44.77×. Since the CREST algorithm cannot easily be parallelized, we ran SHEAR independently on each chromosome using an Intel Xeon 2.66 GHz processor. For this data set, SHEAR was able to detect SVs and generate most chromosomes in only a few hours each, and only two chromosomes (chr1 and chr10) took more than a day. Results for runtime and number of SVs detected as part of the assembly can be seen in Table S4 in Additional file 1. We observed that CREST’s SV detection accounts for a majority of the runtime in most cases. Since SHEAR is designed to work with any base pair resolution SV detection algorithm, future algorithms (i.e. especially algorithms that are parallelizable) can further improve SHEAR’s efficiency.

This analysis was intended mainly to demonstrate SHEAR’s ability to scale up to the size of whole-genome sequencing data sets, however there were also a few noteworthy findings in the results. When compared with a list of 70 large deletions in coding sequences determined by read-depth analysis published by Ju *et al.*[24] for this same data set, there were eight deletions predicted by SHEAR that had breakpoints within 30 bp of a corresponding prediction from Ju *et al*. SHEAR’s heterogeneity estimations for these eight SVs were also mostly consistent with their observed read-depth ratios from Ju *et al.*, with an average absolute error of 9.87 (see Table S5 in Additional file 1). Since we do not have access to the original sample tissue for this data set, it was not possible to do a thorough validation of these results such as was done for the tumor cell line data discussed previously, but the consistency in both breakpoint coordinates and heterogeneity estimation for these select SVs is encouraging. The analysis from Ju *et al.* for this data set was limited to large deletions in coding sequences, so it is also likely that SHEAR may have found additional valid SVs not present in the Ju *et al.* predictions. We also compared SHEAR’s results with genes from the Catalogue of Somatic Mutations in Cancer (COSMIC) [25] and found thirteen SVs that overlapped with genes related to cancer-causing SVs listed in COSMIC (see Table S6 in Additional file 1). Interestingly, one of these SVs was a KIAA1462-KIF5B fusion that was also found by Ju *et al.* in transcriptome data from this same sample, but not previously found in this whole-genome sequencing data set.

## Limitations and future work

The proposed methods contain some inherent limitations and directions for future work:

First, even if the alignment is done perfectly, with no sequencing errors and correct soft-clipping, the estimated heterogeneity level cannot fully account for random fluctuations between the coverage of the reference-like DNA and the variant DNA in the sample. This uncertainty can always be minimized by increasing the depth of the sequencing to reduce the variation in these coverage level fluctuations. Future development for SHEAR will include reporting confidence intervals for heterogeneity estimations to help quantify this uncertainty.

Second, we limit the results and methods discussed in this paper to small, intra-chromosomal SV types for proof of concept in leveraging SV predictions to generate personal genomic sequence and estimating heterogeneity by comparing counts of spanning and soft-clipped reads. We will expand our methods to be more thorough in future versions of SHEAR. Specifically, support for both intra- and inter-chromosomal translocations will be added, as well as support for moderate-sized insertions and more complex variants consisting of overlapping SVs. We will also look to improve upon the assembly component of SHEAR by automatically generating multiple personal genomic sequences using phasing information derived from our estimated heterogeneity levels of variants.

Third, we will explore the possibility of applying SHEAR to other kinds of heterogeneous sequencing data sets, such as pooled population samples or metagenomic samples that can share a similar reference sequence. We believe that SHEAR’s ability to quantify the heterogeneity percentage of predicted SVs makes it an ideal tool to help analyze these types of data sets.

Finally, future work will explore the possibility of using alternative alignment algorithms and SV predictors in the SHEAR pipeline. Newer alignment programs such as GSNAP [26] and BWA-MEM [27] may offer stronger capabilities for proper soft-clipping alignment, and we will investigate how the incorporation of these programs may improve the first part of the SHEAR pipeline. For SV prediction, CREST was shown to work well in the experiments presented here, but any base pair resolution SV detection algorithm would also be compatible as well, such as the more recently developed PRISM [18] or DELLY [28]. Since new SV detection algorithms are being developed regularly, the SHEAR pipeline could be naturally enhanced with each new breakthrough in SV detection approaches.

## Conclusions

We have developed SHEAR, a tool that predicts SVs, accounts for heterogeneous variants by estimating their representative percentages, and generates personal genomic sequences to be used for downstream analysis. Our results demonstrate how SHEAR offers advantages over competing approaches for simulated data sets, tumor cell line data, and whole-genome tumor sequencing data in terms of computational efficiency, tandem duplication events, and an ability to handle heterogeneous sequencing samples. The local realignment component of SHEAR is shown to fix errant soft-clipping and thus improve the accuracy and confidence of SV predictions in comparison with running CREST by itself, as well as improve the accuracy of our heterogeneity estimations. SHEAR is an ideal tool for detecting SVs and generating personal genomic sequences when dealing with heterogeneous sequencing samples.

## Availability and requirements

-  **Project name:** SHEAR
-  **Project home page:**http://vk.cs.umn.edu/SHEAR
-  **Operating system(s):** Unix-like (Linux, Mac OSX, etc.)
-  **Programming language:** Java
-  **Other requirements:** CREST, BWA, Samtools, Picard, GATK
-  **License:** GNU GPL v3
-  **Any restrictions to use by non-academics:** None

## Electronic supplementary material

Additional file 1: **Supplementary Figures and Tables.** Document contains the following Supplementary Figures and Tables: Figure S1 Verified SVs from *AR* locus in CWR-R1 cell line. Figure S2 Alignment at breakpoints for SV #2 from CWR-R1 cell line. Table S1 Correctly detected SVs for simulated data at 1000 × coverage under varying levels of heterogeneity. Table S2 Correctly detected SVs for simulated data at 60% heterogeneity under varying levels of coverage. Table S3 Summary of performance for SHEAR versus standalone CREST on simulated data sets. Table S4 Summary of SHEAR results for whole-genome tumor sequencing data. Table S5 Deletions predicted by both SHEAR and read-depth approaches for whole-genome tumor sequencing data. Table S6 SHEAR SV predictions overlapping with COSMIC genes for whole-genome tumor sequencing data. (PDF 722 KB)

## Abbreviations

ADT: Androgen depletion therapy
*AR*: Androgen receptor gene
BP: Base pair
CRPCa: Castration-resistant prostate cancer
GATK: Genome analysis toolkit
GHz: Gigahertz
indel: Insertion/deletion
kbp: Kilo base pair
MLPA: Multiplex ligation-dependent probe amplification
PCR: Polymerase chain reaction
SHEAR: Sample heterogeneity estimation and assembly by reference
SV: Structural variant.

## References

[1] The ENCODE Project Consortium (2012). An integrated encyclopedia of DNA, elements in the human genome. Nature.

[2] Li H, Durbin R (2009). Fast and accurate short read alignment with Burrows-Wheeler transform. Bioinformatics.

[3] Langmead B, Trapnell C, Pop M, Salzberg SL (2009). Ultrafast and memory-efficient alignment of short DNA sequences to the human genome. Genome Biol.

[4] Zerbino DR, Birney E (2008). Velvet: algorithms for de novo short read assembly using de Bruijn graphs. Genome Res.

[5] Li R, Zhu H, Ruan J, Qian W, Fang X, Shi Z, Li Y, Li S, Shan G, Kristiansen K, Li S, Yang H, Wang J, Wang J (2010). De novo assembly of human genomes with massively parallel short read sequencing. Genome Res.

[6] Gnerre S, MacCallum I, Przybylski D, Ribeiro FJ, Burton JN, Walker BJ, Sharpe T, Hall G, Shea TP, Sykes S, Berlin AM, Aird D, Costello M, Daza R, Williams L, Nicol R, Gnirke A, Nusbaum C, Lander ES, Jaffe DB (2011). High-quality draft assemblies of mammalian genomes from massively parallel sequence data. Proc Natl Acad Sci.

[7] Rausch T, Koren S, Denisov G, Weese D, Emde AK, Döring A, Reinert K (2009). A consistency-based consensus algorithm for de novo and reference-guided sequence assembly of short reads. Bioinformatics.

[8] Klein JD, Ossowski S, Schneeberger K, Weigel D, Huson DH (2011). LOCAS — a low coverage assembly tool for resequencing projects. PLoS One.

[9] Schneeberger K, Ossowski S, Ott F, Klein JD, Wang X, Lanz C, Smith LM, Cao J, Fitz J, Warthmann N, Henz SR, Huson DH, Weigel D (2011). Reference-guided assembly of four diverse Arabidopsis thaliana genomes. Proc Natl Acad Sci.

[10] Kim J, Larkin DM, Cai Q, Zhang Y, Ge RL, Auvil L, Capitanu B, Zhang G, Lewin HA, Ma J, Asan (2013). Reference-assisted chromosome assembly. Proc Natl Acad Sci.

[11] Gan X, Stegle O, Behr J, Steffen JG, Drewe P, Hildebrand KL, Lyngsoe R, Schultheiss SJ, Osborne EJ, Sreedharan VT, Kahles A, Bohnert R, Jean G, Derwent P, Kersey P, Belfield EJ, Harberd NP, Kemen E, Toomajian C, Kover PX, Clark RM, Rätsch G, Mott R (2011). Multiple reference genomes and transcriptomes for Arabidopsis thaliana. Nature.

[12] Lunter G, Goodson M (2011). Stampy: a statistical algorithm for sensitive and fast mapping of Illumina sequence reads. Genome Res.

[13] Chen K, Wallis JW, McLellan MD, Larson DE, Kalicki JM, Pohl CS, McGrath SD, Wendl MC, Zhang Q, Locke DP, Shi X, Fulton RS, Ley TJ, Wilson RK, Ding L, Mardis ER (2009). BreakDancer: an algorithm for high-resolution mapping of genomic structural variation. Nature Methods.

[14] Quinlan AR, Clark RA, Sokolova S, Leibowitz ML, Zhang Y, Hurles ME, Mell JC, Hall IM (2010). Genome-wide mapping and assembly of structural variant breakpoints in the mouse genome. Genome Res.

[15] Wang J, Mullighan CG, Easton J, Roberts S, Heatley SL, Ma J, Rusch MC, Chen K, Harris CC, Ding L, Holmfeldt L, Payne-Turner D, Fan X, Wei L, Zhao D, Obenauer JC, Naeve C, Mardis ER, Wilson RK, Downing JR, Zhang J (2011). CREST maps somatic structural variation in cancer genomes with base-pair resolution. Nature Methods.

[16] Abyzov A, Urban AE, Snyder M, Gerstein M (2011). CNVnator: An approach to discover, genotype, and characterize typical and atypical CNVs from family and population genome sequencing. Genome Res.

[17] Zhang J, Wu Y (2011). SVseq: an approach for detecting exact breakpoints of deletions with low-coverage sequence data. Bioinformatics.

[18] Jiang Y, Wang Y, Brudno M (2012). PRISM: pair-read informed split-read mapping for base-pair level detection of insertion, deletion, and structural variants. Bioinformatics.

[19] McKenna A, Hanna M, Banks E, Sivachenko A, Cibulskis K, Kernytsky A, Garimella K, Altshuler D, Gabriel S, Daly M, DePristo MA (2010). The Genome Analysis Toolkit: a MapReduce framework for analyzing next-generation DNA sequencing data. Genome Res.

[20] Li H, Handsaker B, Wysoker A, Fennell T, Ruan J, Homer N, Marth G, Abecasis G, Durbin R, 1000 Genome Project Data Processing Subgroup (2009). The Sequence Alignment/Map format and SAMtools. Bioinformatics.

[21] Picard http://picard.sourceforge.net

[22] Li H, Durbin R (2010). Fast and accurate long-read alignment with Burrows-Wheeler transform. Bioinformatics.

[23] Li Y, Hwang TH, Oseth LA, Hauge A, Vessella RL, Schmechel SC, Hirsch B, Beckman KB, Silverstein KA, Dehm SM (2012). AR intragenic deletions linked to androgen receptor splice variant expression and activity in models of prostate cancer progression. Oncogene.

[24] Ju YS, Lee WC, Shin JY, Lee S, Bleazard T, Won JK, Kim YT, Kim JI, Kang JH, Seo JS (2012). A transforming KIF5B and RET gene fusion in lung adenocarcinoma revealed from whole-genome and transcriptome sequencing. Genome Res.

[25] Forbes SA, Bindal N, Bamford S, Cole C, Kok CY, Beare D, Jia M, Shepherd R, Leung K, Menzies A, Teague JW, Campbell PJ, Stratton MR, Futreal PA (2010). COSMIC: mining complete cancer genomes in the Catalogue of Somatic Mutations in Cancer. Nucleic Acids Res.

[26] Wu TD, Nacu S (2010). Fast and SNP-tolerant detection of complex variants and splicing in short reads. Bioinformatics.

[27] BWA-MEM http://bio-bwa.sourceforge.net

[28] Rausch T, Zichner T, Schlattl A, Stütz AM, Benes V, Korbel JO (2012). DELLY: structural variant discovery by integrated paired-end and split-read analysis. Bioinformatics.

