# Supplementary material for: SHEAR: sample heterogeneity estimation and assembly by reference
Source: BMC Genomics. 2014 Jan 29;15(1):84. doi: 10.1186/1471-2164-15-84 (PMC4007568; doi:10.1186/1471-2164-15-84)
Supplement: Supplementary file 1 — Additional file 1: Supplementary Figures and Tables. Document contains the following Supplementary Figures and Tables: Figure S1 Verified SVs from AR locus in CWR-R1 cell line. Figure S2 Alignment at breakpoints for SV #2 from CWR-R1 cell line. Table S1 Correctly detected SVs for simulated data at 1000 × coverage under varying levels of heterogeneity. Table S2 Correctly detected SVs for simulated data at 60% heterogeneity under varying levels of coverage. Table S3 Summary of performance for SHEAR versus standalone CREST on simulated data sets. Table S4 Summary of SHEAR results for whole-genome tumor sequencing data. Table S5 Deletions predicted by both SHEAR and read-depth approaches for whole-genome tumor sequencing data. Table S6 SHEAR SV predictions overlapping with COSMIC genes for whole-genome tumor sequencing data. (PDF 722 KB) [file 12864_2013_7009_MOESM1_ESM.pdf]

Supplementary Figure 1 - Verified SVs from AR locus in CWR-R1 cell line

(**a**) The locations of the four verified deletions are depicted within the *AR* gene locus. The eight exons are marked with vertical bars, with the 5' and 3' UTRs marked with shorter bars at the ends of the locus. The orientation and order of PCR primers used to verify each SV are shown along the bottom. SV #3 was verified in a previous study and thus PCR validation was not performed on it here. For clarity, some features and positioning on the figure may not be to scale. (**b**) Amplified product from PCR to validate the presence of SV #2. (**c**) Electropherogram peak trace to validate the fusion signature of SV #2. The highlighted bases (i.e. TC) represent microhomology of identical bases on both breakpoint boundaries. (**d**) Amplified product from PCR to validate the presence of SV #4. (**e**) Electropherogram peak trace to validate the fusion signature of SV #4. The highlighted bases (i.e. TGT) represent microhomology of identical bases on both breakpoint boundaries. (**f**) Amplified product from nested PCR to validate the presence of SV #5. The first round of nested PCR using the outermost primers did not reveal any amplified product, but the second round using the interior primers validates the presence of the variant. (**g**) Electropherogram peak trace to validate the fusion signature of SV #5. The highlighted bases (i.e. AGCCAGCA) represent microhomology of identical bases on both breakpoint boundaries.

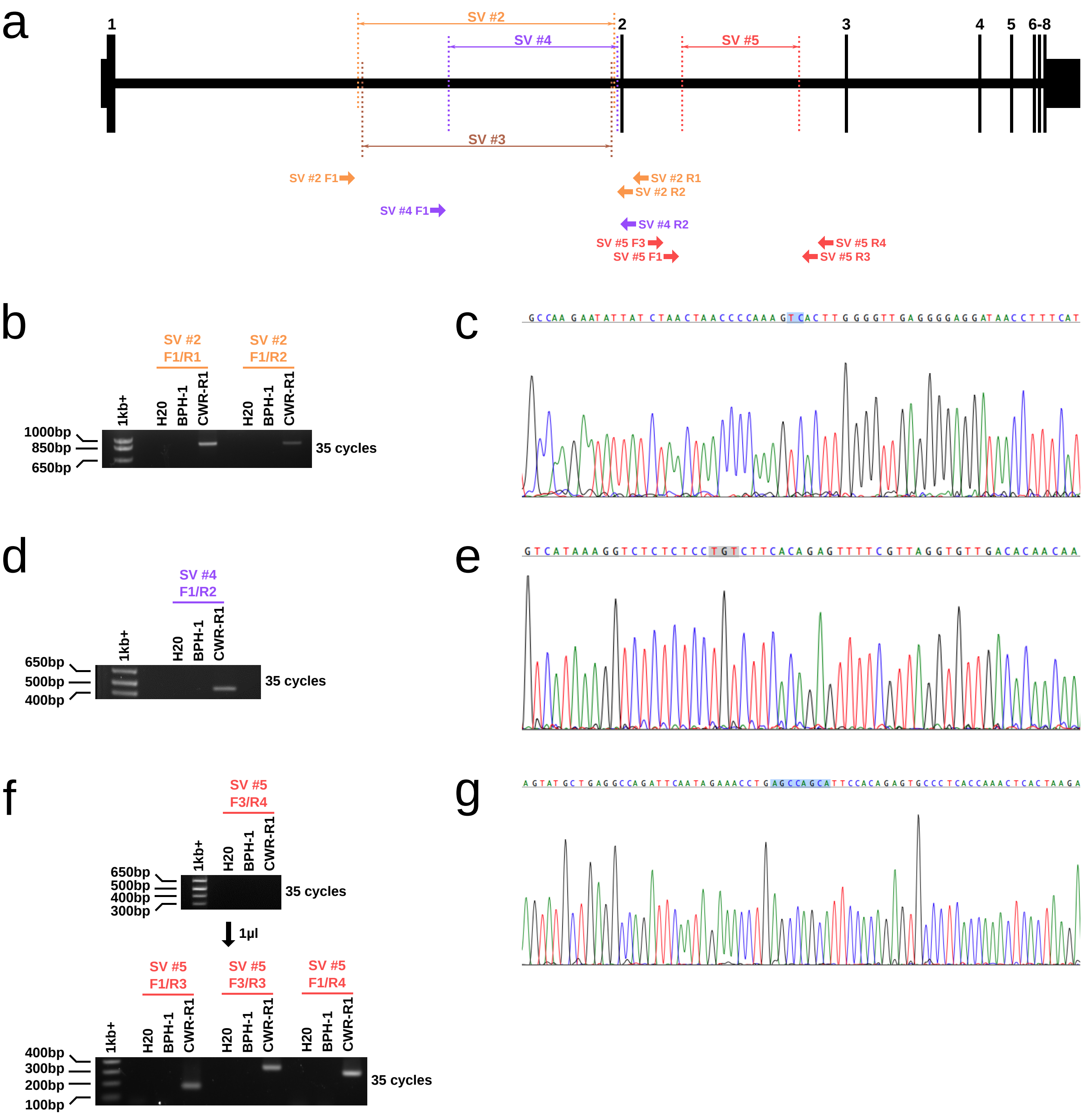
a
1
2
3
4
5
6-8
SV #2
SV #4
SV #5
SV #3
SV #2 F1
SV #2 R1
SV #2 R2
SV #4 F1
SV #4 R2
SV #5 F3
SV #5 F1
SV #5 R4
SV #5 R3
b
SV #2 F1/R1
SV #2 F1/R2
1kb+
H20
BPH-1
CWR-R1
1000bp
850bp
650bp
35 cycles
c
d
SV #4 F1/R2
650bp
500bp
400bp
35 cycles
e
f
SV #5 F3/R4
650bp
500bp
400bp
300bp
35 cycles
1μl
SV #5 F1/R3
SV #5 F3/R3
SV #5 F1/R4
400bp
300bp
200bp
100bp
35 cycles
g

Supplementary Figure 2 - Alignment at breakpoints for SV #2 from CWR-R1 cell line

A portion of the alignment of CWR-R1 sequencing data is shown at the two breakpoints for SV #2, located within the *AR* gene, with reference sequence shown along the bottom. At the left breakpoint (chrX:66,812,839) there are 702 reads that are soft-clipped and 3,094 reads that span the breakpoint. At the right breakpoint (chrX:66,861,669) there are 412 reads that are soft-clipped and 2,306 reads that span the breakpoint. Using SHEAR's heterogeneity estimation scheme, these numbers predict a variant heterogeneity level of 29.21%. Note that there is a microhomology of 2 bp (TC) that borders both breakpoints, and the fusion of the two sides will only contain one copy of these two nucleotides. Visualization performed using IGV (http://www.broadinstitute.org/igv/).

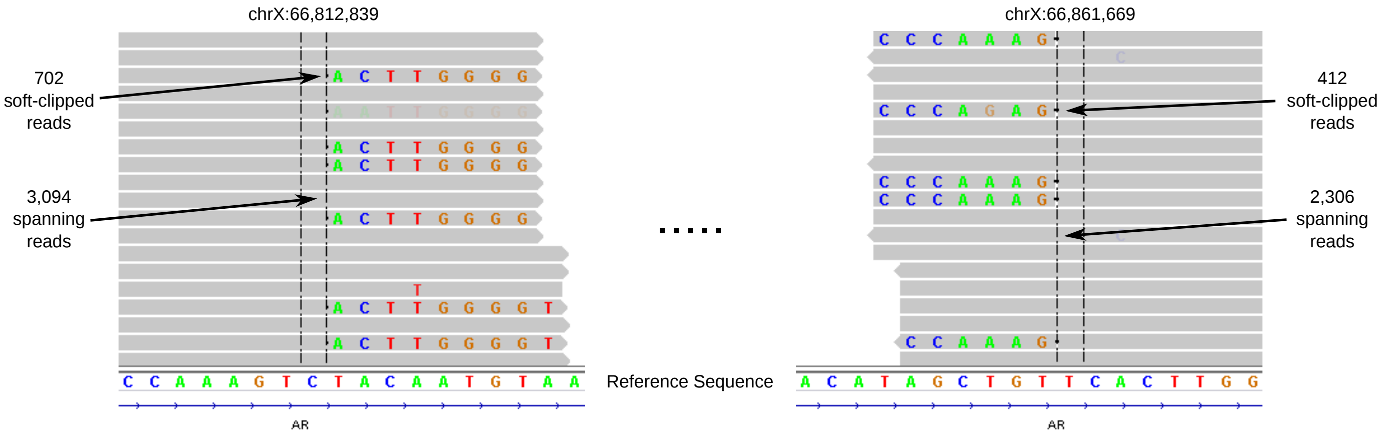

Table S1 - Correctly detected SVs for simulated data at 1000× coverage under varying levels of heterogeneity

All simulations are done on a 70,000 bp portion of chromosome 15 after introducing deletions and tandem duplications of sizes 150 bp, 1000 bp, and 30,000 bp, each over 10 different iterations, for a total of 30 different deletion events, and 30 different tandem duplication events.

	**Deletions**		**Tandem Duplications**	
**Variant Percent**	**SHEAR**	**IMR/DENOM**	**SHEAR**	**IMR/DENOM**
**20%**	29 / 30	0 / 30	28 / 30	0 / 30
**40%**	29 / 30	0 / 30	28 / 30	0 / 30
**60%**	29 / 30	0 / 30	28 / 30	0 / 30
**80%**	29 / 30	0 / 30	28 / 30	0 / 30
**90%**	29 / 30	0 / 30	28 / 30	0 / 30
**100%**	29 / 30	17 / 30	28 / 30	0 / 30

Table S2 - Correctly detected SVs for simulated data at 60% heterogeneity under varying levels of coverage

All simulations are done on a 70,000 bp portion of chromosome 15 after introducing deletions and tandem duplications of sizes 150 bp, 1000 bp, and 30,000 bp, each over 10 different iterations, for a total of 30 different deletion events, and 30 different tandem duplication events.

	Deletions		Tandem Duplications	
**Depth**	**SHEAR**	**IMR/DENOM**	**SHEAR**	**IMR/DENOM**
**10×**	7 / 30	0 / 30	7 / 30	0 / 30
**20×**	26 / 30	0 / 30	24 / 30	0 / 30
**30×**	29 / 30	0 / 30	26 / 30	0 / 30
**50×**	29 / 30	0 / 30	27 / 30	0 / 30
**100×**	29 / 30	0 / 30	28 / 30	0 / 30
**500×**	29 / 30	0 / 30	28 / 30	0 / 30
**1000×**	29 / 30	0 / 30	28 / 30	0 / 30

Table S3 - Summary of performance for SHEAR versus standalone CREST on simulated data sets

This table compares the SV predictions for our simulated data sets between SHEAR's results and CREST's results. SHEAR uses CREST as its internal SV detection algorithm, but also uses a targeted local realignment to fix incorrect soft-clipping in order to improve the accuracy and confidence of CREST's SV predictions. All simulations are done on a 70,000 bp portion of chromosome 15 after introducing deletions and tandem duplications of sizes 150 bp, 1000 bp, and 30,000 bp, each over 10 different iterations, for a total of 30 different deletion events, and 30 different tandem duplication events. Sequencing data was simulated for each of these synthetic sequences, with heterogeneity percentage varying over six settings (i.e. 20%, 40%, 60%, 80%, 90%, and 100% of reads from the variant sequence) and overall average coverage varying over seven settings (10×, 20×, 30×, 50×, 100×, 500×, and 1000×) for a total of 2520 SVs present in the simulated data sets. There is one SV that standalone CREST detects which SHEAR misses, one SV that SHEAR detects and standalone CREST misses, and 2072 SVs that are found by both. Of the 2072 SVs found by both, CREST has more accurate breakpoints for three of them while SHEAR has more accurate breakpoints for 502 of them due to SHEAR's targeted local realignment to fix incorrect soft-clipping.

**SVs predicted by CREST:**	2073 / 2520
**SVs predicted by SHEAR:**	2073 / 2520
**SVs predicted by both:**	2072 / 2520
More accurate breakpoints with CREST:	3 / 2072
More accurate breakpoints with SHEAR:	502 / 2072
Same breakpoints:	1567 / 2072

Table S4 - Summary of SHEAR results for whole-genome tumor sequencing data

SHEAR was run on each chromosome independently on an Intel Xeon 2.66 GHz processor. Whole-genome sequencing data had an average depth of coverage of 44.77×. The number of SVs reported includes all SVs predicted by SHEAR, although some may be overlapping. In these cases, the SV with a higher prediction confidence (i.e. more supporting soft-clipped reads) was used.

Chromosome	Runtime (Minutes)	# SVs
**1**	4009.60	149
**2**	740.13	159
**3**	1146.30	111
**4**	609.47	161
**5**	201.58	117
**6**	142.75	144
**7**	243.03	118
**8**	180.80	97
**9**	271.25	78
**10**	7190.87	100
**11**	403.63	39
**12**	118.75	70
**13**	95.98	59
**14**	76.92	50
**15**	91.40	56
**16**	494.5	30
**17**	193.4	35
**18**	147.23	52
**19**	303.60	26
**20**	72.28	32
**21**	54.08	22
**22**	42.80	15
**X**	106.93	30
**Y**	1375.00	1

Table S5 - Deletions predicted by both SHEAR and read-depth approaches for whole-genome tumor sequencing data

SHEAR SV predictions from whole-genome sequencing data from a lung cancer patient were compared against a previously published list of large deletions in coding sequences determined via read-depth analysis for the same sample [1]. Breakpoint coordinates for both SHEAR and the read-depth analysis are listed for each deletion, as well as SHEAR's estimated heterogeneity percentage. For the read-depth analysis, the read-depth ratio relative to the neighboring regions is used to give a rough approximation of the heterogeneity percentage of the deletion as measured by the read-depth analysis (i.e. $VariantPercent = 1 - RelativeRDRatio$).

Read-Depth Analysis (from Ju *et al.*)			SHEAR Predictions	
**Location**	**Relative RD Ratio**	**Variant Percent**	**Location**	**Variant Percent**
chr1:108534856-108538779	0.3859	61.41%	chr1:108534849-108538774	78.05%
chr1:143804306-143808442	0.2770	72.30%	chr1:143804306-143808438	66.67%
chr2:110210032-111169571	0.2776	72.24%	chr2:110210044-111169574	56.25%
chr3:131246078-131289429	0.0409	95.91%	chr3:131246073-131289435	100.00%
chr11:5741122-5765879	0.4469	55.31%	chr11:5741152-5765858	63.41%
chr11:7673481-7673805	0.0000	100.00%	chr11:7673492-7673795	100.00%
chr16:69535301-69537421	0.6322	36.78%	chr16:69535301-69537391	56.00%
chr22:22604141-22641298	0.5621	43.79%	chr22:22606437-22609227	90.91%

Table S6 - SHEAR SV predictions overlapping with COSMIC genes for whole-genome tumor sequencing data

SHEAR SV predictions from whole-genome sequencing data from a lung cancer patient were compared against genes with SVs listed in the Catalogue of Somatic Mutations in Cancer (COSMIC) [2]. SV type, breakpoint coordinates, SHEAR's estimated heterogeneity percentage, and the set of overlapping cancer genes from COSMIC are listed for each predicted SV.

SV Type	Breakpoints		Variant Percent	Overlapping COSMIC Genes
Deletion	chr1:143665972	chr1:143666718	40.00%	PDE4DIP
Deletion	chr1:143738104	chr1:143738409	55.32%	PDE4DIP
Deletion	chr2:29,852,732	chr2:29,852,803	100.00%	ALK
Deletion	chr2:22,346,8436	chr2:22,347,0915	100.00%	ACSL3
Deletion	chr3:52684890	chr3:52685207	66.67%	PBRM1
Deletion	chr5:38546765	chr5:38546821	100.00%	LIFR
Deletion	chr6:167338101	chr6:167338160	60.00%	FGFR1OP
Deletion	chr12:11917683	chr12:11918420	54.55%	ETV6
Deletion	chr13:38926482	chr13:38926791	100.00%	LHFP
Deletion	chr17:53042811	chr17:53044913	100.00%	MSI2
Translocation	chr1:36505837	chr1:36507229	25.00%	THRAP3
Tandem Duplication	chr10:30387994	chr10:32348629	30.00%	KIF5B
Tandem Duplication	chr12:34263877	chr12:126216940	100.00%	ALDH2, ATF1, BCL7A, BTG1, DDIT3, HMGA2, HOXC11, HOXC13, LRIG3, MDM2, NACA, WIF1

References

1. Ju YS, Lee WC, Shin JY, Lee S, Bleazard T, Won JK, Kim YT, Kim JI, Kang JH, Seo JS: **A transforming KIF5B and RET gene fusion in lung adenocarcinoma revealed from whole-genome and transcriptome sequencing**. *Genome Research* 2012, **22**(3):436–445.

2. Forbes SA, Bindal N, Bamford S, Cole C, Kok CY, Beare D, Jia M, Shepherd R, Leung K, Menzies A, Teague JW, Campbell PJ, Stratton MR, Futreal PA: **COSMIC: mining complete cancer genomes in the Catalogue of Somatic Mutations in Cancer**. *Nucleic Acids Research* 2010, **39**(Databse issue):D945–D950.
